# Robot-assisted enucleation of gigantic liver hemangiomas: Case series of 3 patients

**DOI:** 10.1016/j.ijscr.2019.06.033

**Published:** 2019-06-21

**Authors:** Pier Cristoforo Giulianotti, Roberto Bustos, Valentina Valle, Gabriela Aguiluz, Yevhen Pavelko, Eduardo Fernandes

**Affiliations:** Division of General, Minimally Invasive and Robotic Surgery, Department of Surgery, University of Illinois at Chicago, 840 S. Wood Street, Suite 435E (MC 958), Chicago, IL, 60612, USA

**Keywords:** Hemangioma, Liver, Robotic, Enucleation, Resection, Case series

## Abstract

**Introduction:**

Liver hemangiomas are benign liver lesions that require surgical excision when large and symptomatic. Often, these tumors are removed through large anatomical resections which sacrifice more liver parenchyma than needed. Tumor enucleation, which takes advantage of a digitoclasia-like technique along the plane between the tumor pseudo-capsule and liver parenchyma, poses challenges when performed laparoscopically. We propose a parenchymal sparing, minimally invasive, robotic-assisted technique to remove liver hemangiomas.

**Presentation of cases:**

Three male patients with symptomatic hemangiomas were treated in our center between 2015 and 2018. The lesions were located in segment II, III and segment IV-V respectively. Robotic-assisted hemangioma enucleation was accomplished successfully in all three patients. The procedures were performed with a parenchyma-sparing intent and a formal segmentectomy was not required. There were no conversions to open surgery.

**Discussion:**

The robotic platform provides a powerful tool in the enucleation of liver hemangiomas. The enhanced vision and the superior suturing ability allow to develop safely the plane between the tumor pseudo-capsule and the liver parenchyma, with outstanding selective control of all the vascular supply and drainage encountered during the dissection. Furthermore, precision of the hilar dissection allows selective lobar or sectorial arterial control which helps minimize intraoperative bleeding.

**Conclusion:**

In our experience, robot-assisted enucleation of liver hemangiomas offers low morbidity, fast recovery, excellent cosmetic results, and it could become a therapeutic option when the location of the hemangioma allows this approach.

## Highlights

• The key aspect of this technique is to selectively control the arterial inflow.
• Mass enucleation represents the most challenging part of the procedure.
• ICG allows identifying the surgical plane between the mass and normal parenchyma.
• The robotic platform allows to replicate the enucleation performed in open surgery.

## 1 Introduction

Hemangiomas are the most common benign hepatic tumors, with a prevalence that ranges from 1% to 20% [1]. Often diagnosed incidentally, they can occasionally grow and cause compressive symptoms [2]. Previous reports described robot-assisted formal segmentectomies or lobectomies [3,4], but not enucleations. This technique has the main advantage of increasing parenchymal preservation and decreasing intra-operative bleeding, avoiding some of the technical challenges and risks of formal segmentectomies or other non-anatomical resections. This work has been reported in line with the PROCESS guidelines [5].

## 2 Methods

This retrospective study involved 3 patients who underwent robot-assisted hemangioma enucleation, performed by PCG at our academic institution between July 2015 and November 2018 and was approved by the Institutional Review Board.

## 3 Surgical technique

**Fig. 1:**
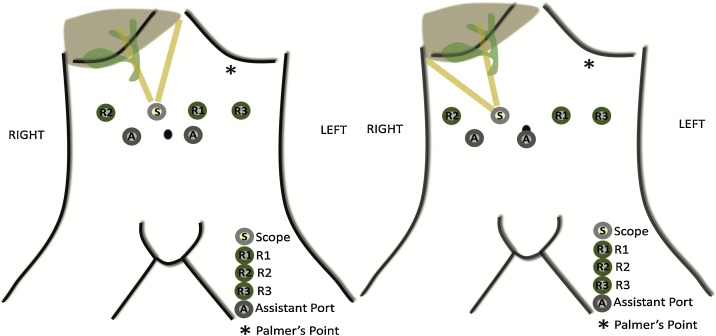
Port placement. Legend: port placement for left lobe hemangiomas (left); port placement for right lobe hemangiomas (right).

The patient is placed supine with parted legs and in a 20 degrees reverse Trendelenburg position. Exploratory laparoscopy and intraoperative ultrasound are performed for lesion localization and port placement strategy. The configuration is shown in Fig. 1 [6]. The falciform ligament is divided with the Harmonic shears (Ethicon Endo-Surgery, Inc., Cincinnati, OH) to improve exposure. Indocyanine green (ICG) can be injected to assess the vascular pattern of the mass. ICG uptake of hemangiomas is characterized by a faster and brighter enhancement compared to normal liver parenchyma and a more rapid ‘wash out’. This pattern of fluorescence allows the identification of the correct surgical plane between the mass and normal parenchyma. Smaller satellite lesions can be found upon ICG injection.

The key aspect of this technique is to selectively control the arterial inflow to the lesion. Arterial clamping usually is done at lobar level in the hilum.

Keeping a low central venous pressure during the parenchymal transection is advised, as it facilitates bleeding control.

Mass enucleation represents the most challenging part of the procedure. The identification of a plane between the hemangioma’s pseudo-capsule and the liver parenchyma is almost invariably possible. When hemangiomas arise from the liver surface, the surgical plane is of relatively easy identification. Hemangiomas located deep into the liver require dissection of normal parenchyma prior to enucleation. This poses greater challenges to the surgeon in terms of resection margin adequacy and sparing of the parenchyma. Once the correct plane is identified, small connecting veins are found between the hemangioma and normal liver tissue. These can be coagulated with bipolar forceps or suture ligated with 4-0 or 5-0 Prolene.

Following enucleation, the arterial bulldog clamp is removed and the central venous pressure is increased to test and further secure the hemostasis. Fibrin glue is applied to the surface of the liver. A Jackson-Pratt drain is left by the enucleation site and the specimen is retrieved enlarging one of the port sites or a Pfannenstiel incision depending on size.

## 4 Case 1

**Fig. 2:**
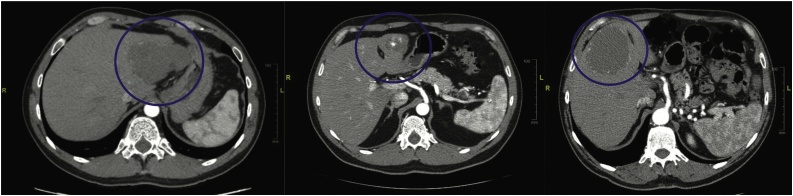
Pre operatory CT scan showing liver hemangioma. Legend: Case 1 CT scan (left); Case 2 CT scan (center); Case 3 CT scan (right).

**Fig. 3:**
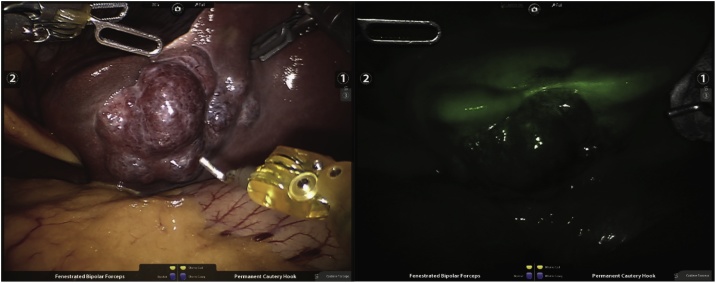
Superficial view of the liver mass. (Case 1). Legend: Image comparison: bright light (left) vs ICG fluorescence (right).

A 42-year-old man was seen in our outpatient department complaining of moderate left upper quadrant abdominal pain. He was known to have a liver hemangioma diagnosed 1.5 years prior. Double helical CT scan revealed an 8.2 x 5.8 cm (Fig. 2) vascular lesion within segments II and III of the liver with peripheral nodular enhancement. The surgery was performed according to the technique described above. The vascular pattern of the hemangioma could be differentiated from the one of the liver using ICG (Fig. 3). Patient’s outcomes are summarized in Table 1.

**Table 1:** Summary of patient demographics and outcomes.

Case	Gender	Age	BMI (kg/m^2^)	ASA	Liver Segment	Procedure	Tran.	OT (min)	EBL (mL)	Morb.	Mort.	LOS (days)	Pathology
1	M	42	24.87	1	II	Robotic enucleation of angioma	0	146	100	0	0	3	Hemangioma
2	M	45	27.36	2	III	Robotic enucleation of angioma	0	121	50	0	0	4	Cavernous hemangioma
3	M	61	25.49	2	IV-V	Robotic enucleation of angioma + Cholecystetomy	2 units	193	600	PE	0	5	Hemangioma

BMI = Body Mass index; ASA = American Society of Anesthesiologists score; Tran. = Transfusion; OT = Operative time; EBL = Estimated blood loss; Morb. = Morbidity; Mort. = Mortality; LOS = length of stay; PE = Pulmonary embolism.

## 5 Case 2

**Fig. 4:**
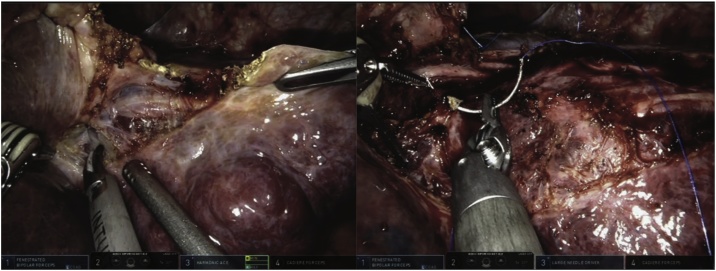
Hemangioma enucleation (Case 2). Legend: Resection using Harmonic shears (left); Small vessel transection in between sutures (right).

A 44-year-old man presented to our clinic with long-standing left-upper quadrant pain. CT scan revealed a 3.9 x 3.5 cm segment III hemangioma (Fig. 4). Positioning, trocar placement and surgical technique previously described. Outcomes are summarized in Table 1.

## 6 Case 3

**Fig. 5:**
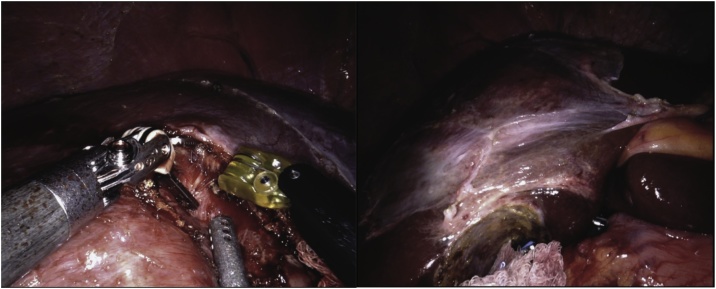
Deep Giant hemangioma involving the majority of segment IV (Case 3). Legend: superficial view (left); Hemangioma detachment following the pseudo capsule plane (right).

A 62-year-old man with a known diagnosis of liver hemangioma, presented to our outpatient department with right-upper quadrant pain. CT scan imaging showed a 9.1 × 7 x 2.2 cm mass occupying segments IVa and V. Prior to surgery, the patient underwent selective arterial embolization in the attempt to decrease the size of the lesion. Trocar placement was similar to the one showed in Fig. 1. Intraoperative US scanning showed that the deeper part of the hemangioma was very close to the bifurcation of the portal elements. Liver hilum was dissected, and a bulldog was applied to the common hepatic artery. After the falciform ligament was taken down, dissection started between the segments III and IVB, on the right side of the falciform (Fig. 5). The operation followed using the same technique previously described. Outcomes are summarized in Table 1. The patient had an uneventful post operatory course and was discharged on POD 5. One day after discharge, patient was readmitted with a pulmonary embolism which was successfully treated with subcutaneous low molecular weight heparin.

## 7 Discussion

Liver hemangiomas are usually managed expectantly with serial imaging follow-ups [2]. Surgical indications for this pathology are still a matter of debate [7]. The current consensus to surgical resections pivots around the size of the lesion (10 cm or above), the presence of symptoms, complications (although bleeding and rupture are extremely rare events) and the presence of consumptive coagulopathy (Kasabach-Merritt syndrome) [8].

Surgery for these lesions is challenged by the high risk of bleeding, which discourages many surgeons from performing these resections in a minimally invasive fashion [7]. Based on our experience, a laparoscopic approach should be favored in patients with solitary lesions located anterolaterally (segments II to VI) and between 5 and 10 cm in size. Some authors have reported laparoscopic excision of liver hemangiomas through a formal lobectomy or segmentectomy [9,10], but very few reported laparoscopic enucleations [11]. With regards to the laparoscopic enucleations with selective arterial clamping [12], the mainstay of this technique lies within an accurate hilar dissection and fast and accurate liver parenchymal suturing. Enucleation and arterial clamping are safely executed on a robotic platform.

In the open approach, a delicate detachment of the tumor from the pseudo-capsule can be done digitally, proceeding with blunt movements. As the dissection proceeds, tumor feeding and draining vessels are ligated as they are encountered. This technique based on tactile feedback cannot be replicated in minimally invasive surgery. Texture, consistency, and pulsatility of the tumor cannot be appreciated on the robotic platform. Nevertheless, the high-quality imaging provided by the camera system allows developing a “virtual haptic feedback”. Magnified visualization of minimal changes resulting from the interaction of the instruments with the tissues, enables the surgeon to “virtually feel” the tissue resistance. This in some way replaces the lack of tactile feedback. Such ‘sixth sense’ for tissue consistency, however, is developed after a significant experience at the console.

To our knowledge, this is the first report of robotic liver hemangioma enucleation. The microsurgical abilities of the robot allow a delicate dissection with an accurate detachment of the tumor from its pseudo-capsule. Selective suturing of small vascular branches connected to the tumor is possible with the robotic platform, replicating the enucleation performed in open surgery but in a minimally invasive fashion. Furthermore, ICG allows the recognition of vascular patterns and anomalies intraoperatively [13,14].

## 8 Conclusion

Robotic-assisted surgical enucleation of liver hemangiomas is safe and feasible. Further studies involving more patients are needed to expand the application of robotic surgery for these procedures.

## Conflicts of interest

Pier Cristoforo Giulianotti has a consultant agreement with Covidien Medtronic and Ethicon Endosurgery, and he also has an institutional agreement (University of Illinois at Chicago) for training with Intuitive. Roberto Bustos, Valentina Valle, Gabriela Aguiluz, Yevhen Pavelko, and Eduardo Fernandes have no conflicts of interest or financial ties to disclose.

## Funding

This research was not conducted with any specific grants from funding agencies in the public, commercial, or not-for-profit sectors.

## Ethical approval

This study has been approved by the Institutional Review Board at our institution.

## Consent

Written informed consent was obtained from the patients for publication.

## Author’s contribution

PCG: data analysis and interpretation, writing and revision.

RB: study design, data collection, and writing.

VV, GA, YP: collection and assembly of data.

EF: writing and revision.

## Registration of research studies

Study registered with Research Registry UIN researchregistry4777.

## Guarantor

Pier Cristoforo Giulianotti.

Roberto Bustos.

Eduardo Fernandes.

## Provenance and peer review

Not commissioned, externally peer-reviewed.
